# Comprehensive Molecular Screening by Next Generation Sequencing of Gastrointestinal Stromal Tumors (GISTs): In Silico Analysis and Classification of Rare *KIT* Exon 11 Mutations

**DOI:** 10.1002/cam4.71430

**Published:** 2025-12-08

**Authors:** Maria Grazia Rodriquenz, Barbara Pasculli, Michelina Rendina, Francesco Petrizzelli, Tommaso Mazza, Antonio Petracca, Bartolomeo Augello, Michelina Coco, Teresa Balsamo, Anna Troiano, Magda Zanelli, Evaristo Maiello, Paolo Graziano, Paola Parente, Paola Parrella

**Affiliations:** ^1^ Unit of Oncology Fondazione IRCCS Casa Sollievo Della Sofferenza Foggia Italy; ^2^ Laboratory of Oncology Fondazione IRCCS Casa Sollievo Della Sofferenza Foggia Italy; ^3^ Unit of Bioinformatics Fondazione IRCCS Casa Sollievo Della Sofferenza Foggia Italy; ^4^ Computational Biology and Bioinformatics Unit Fondazione Policlinico Universitario A. Gemelli Rome Italy; ^5^ Medical Genetics Unit Fondazione IRCCS Casa Sollievo Della Sofferenza Foggia Italy; ^6^ Pathology Unit Azienda USL‐IRCCS di Reggio Emilia Reggio Emilia Italy; ^7^ Unit of Pathology, Fondazione IRCCS Casa Sollievo Della Sofferenza Foggia Italy; ^8^ Department of Radiological, Oncological and Pathological Sciences Sapienza University of Rome Italy

**Keywords:** gastrointestinal stromal tumors (GISTs), *KIT*, Next Generation Sequencing (NGS), *PDGFRA*, *SDH* genes, structural bioinformatics

## Abstract

**Introduction:**

Gastrointestinal stromal tumors (GISTs) originate from mesenchymal precursor cells of the gastrointestinal wall and account for approximately 3% of all gastrointestinal malignancies. In adults, these tumors most commonly harbor mutually exclusive activating mutations in *KIT*, *PDGFRA*, *BRAF*, or *SDH*‐family genes. *KIT* and *PDGFRA* mutations are well‐established predictive biomarkers for response to tyrosine kinase inhibitors (TKIs). The advent of Next Generation Sequencing (NGS) has accelerated the identification of novel genetic alterations, improving disease characterization, providing prognostic and predictive information, and enabling detection of rare germline variants.

**Methods:**

We conducted a retrospective analysis of 31 patients with GIST to evaluate the implementation of NGS testing and the interpretation of pathogenicity in real‐life clinical practice. Identified mutations were compared with publicly available databases, and rare KIT exon 11 variants underwent computational modeling to assess their impact on protein conformation and imatinib interaction. Germline testing was performed when indicated.

**Results:**

In addition to common primary and secondary mutations in *KIT* and *PDGFRA*, three patients carried rare *KIT* exon 11 variants not previously classified in public databases: p.Gln556del (upstream of codons 557/559) and p.Asn566_Pro573del and p.Val569_Leu576_del (downstream), all located within the juxtamembrane (JM) domain. One patient harbored an S*DHB* c.287‐1G⟩C alteration resulting in aberrant splicing, also detected in the germline. The structural modeling predicted that all rare KIT exon 11 variants affected protein conformation and influenced imatinib interaction. Clinically, all three variants were associated with response to imatinib and met ACMG‐AMP criteria for pathogenicity as well as ASCO‐CAP tier 1 classification.

**Conclusions:**

Our findings highlight the clinical relevance of integrating NGS into routine GIST management, particularly for the identification and interpretation of rare genetic variants. The study underscores the importance of data sharing and collective variant annotation to support accurate molecular classification, prognostic assessment, and therapeutic decision‐making for individual patients.

## 1 Introduction

Gastrointestinal stromal tumors (GISTs) are the most common mesenchymal tumors of the gastrointestinal tract, accounting for approximately 3% of all gastrointestinal neoplasms [1]. In adults, the majority of GISTs (~85%) harbor mutually exclusive mutations in either *KIT* or *PDGFRA* genes that lead to the constitutive activation of corresponding tyrosine kinase receptors 2 [2]. In particular, activating mutations in the *KIT* gene are reported in 75%–80% of GISTs, while *PDGFRA* mutations are found in 5%–10% of GISTs. The remaining 15% of adult GISTs and about 85% of pediatric GISTs do not show any *KIT* or *PDGFRA* mutation and may carry alterations in the genes encoding for succinate dehydrogenase (SDH) complex or in *BRAF*, *PIK3CA*, *RAS*, *NTRK* genes [3, 4, 5, 6].

Different mutations in the *KIT* gene are associated with variable responses to specific tyrosine kinase inhibitors (TKIs). Primary mutations most frequently occur in exons 9 and 11, with exon 11 mutations showing greater sensitivity to imatinib, and correlating with improved progression‐free survival (PFS) and overall survival (OS). In contrast, exon 9 mutations, more commonly observed in small bowel GISTs, may require higher doses of imatinib for optimal response [7, 8]. However, secondary *KIT* mutations, particularly those affecting exons 13, 14, 17, and 18, are associated with resistance to imatinib, limiting its long‐term effectiveness. In the second‐line setting and beyond, sunitinib has demonstrated superior activity over imatinib in inhibiting *KIT* mutants harboring double mutations in exons 9, 11, 13, or 14. Meanwhile, agents such as regorafenib, dasatinib, nilotinib, and sorafenib have shown substantial efficacy in targeting secondary exon 17 mutations, effectively inhibiting *KIT* phosphorylation and downstream signaling [7, 8]. Notably, in a subgroup analysis of the INTRIGUE trial, ripretinib showed a potential clinical benefit over sunitinib treatment in patients with *KIT* exon 11 mutations combined with exon 17/18 [9, 10]. Mutations in *PDGFRA* are mainly found in exons 18 and 12 and rarely occur in exon 14. Exon 18 encodes the activation loop and represents approximately 80% of the *PDGFRA*‐mutated GISTs [3, 4]. A single Arg842Val substitution confers resistance to imatinib, sunitinib, and regorafenib [11]. This is the most common exon 18 mutation, detected in 62.6% of *PDGFRA*‐mutated tumors [3, 4]. Studies have shown that novel tyrosine kinase inhibitors (TKIs), avapritinib and ripretinib, target the PDGFRA Arg842Val mutation in GISTs and provide objective responses and long‐term tumor control [12].

Resistance to imatinib commonly develops within 12–36 months of initiating therapy, largely due to the emergence of secondary *KIT* or *PDGFRA* mutations that alter drug binding affinity. As a result, comprehensive and continuous molecular monitoring, ideally through serial tissue biopsies or liquid biopsy approaches [10, 13] is essential to detect resistance mechanisms in real time. In this context, it becomes increasingly important to accurately classify genetic variants, as this information serves as the foundation for precision oncology and patient‐tailored treatment decisions.

In this study, we present insights derived from our real‐world experience implementing Next Generation Sequencing (NGS) for mutational profiling of gastrointestinal stromal tumors (GISTs) in a retrospective cohort of 31 patients. Consistent with the established molecular landscape, pathogenic mutations in the *KIT* or *PDGFRA* genes were identified in 87% of cases, while one patient exhibited a mutation in the *SDHB* gene. Additionally, secondary resistance mutations in *KIT* were detected in two patients, highlighting the clinical importance of serial molecular monitoring. Among the 20 mutations identified in *KIT* exon 11, three rare variants were detected. In accordance with the guidelines from the American College of Medical Genetics and Genomics and the Association for Molecular Pathology (ACMG‐AMP) [14] and the American Society of Clinical Oncology and College of American Pathologists (ASCO‐CAP) [15], these rare variants underwent rigorous interpretation using established classification criteria and in silico predictive tools to assess their potential pathogenicity and influence on imatinib sensitivity.

## 2 Methods

### 2.1 Samples

We retrospectively analyzed a cohort of 31 patients diagnosed with gastrointestinal stromal tumors (GISTs) from the Pathology Unit at Fondazione IRCCS Casa Sollievo della Sofferenza, all of whom underwent molecular profiling by next‐generation sequencing (NGS). The median age at diagnosis was 60 years (interquartile range [IQR], 50–68), with 16 female (52%) and 15 male (48%) patients. The median follow‐up duration was 25 months (IQR, 20–68). As described in Table 1 and Table S1, [16] patients were affected by localized disease, and five of them were treated with imatinib as adjuvant therapy after surgery. Four patients were affected by locally advanced disease: two received imatinib as neoadjuvant therapy followed by surgery; one developed metastases and was treated with sunitinib as second‐line treatment; one patient was diagnosed before imatinib implementation and received epirubicin and ifosfamide as first‐line metastatic treatment and imatinib as second‐line metastatic therapy. The remaining patient affected by locally advanced disease was lost at follow‐up after surgery. Of the eight patients affected by metastatic disease, diagnosis was performed on tissue biopsy (*n* = 6) or Fine Needle Aspirate Biopsy (FNAB; *n* = 2). Seven patients received imatinib as first‐line treatment, and five of them progressed. Two patients were treated with sunitinib as second‐line treatment. Complete pathologic and clinical data are shown in Table S1.

**TABLE 1:** Patient cohort clinicopathological characteristics.

Variables		*Number of cases*	Percentage
Site	Stomach	20	65%
	Ileum	6	19%
	Jejunum	2	6%
	Peritoneum	1	3%
	Duodenum	1	3
	Small bowel	1	3%
Morphology	Spindle	19	61%
	Epithelioid	5	16%
	Mixed	7	23%
NIH grade	High	5	16%
	Moderate	4	13%
	Low	5	16%
	Very low	3	10%
	Null	2	6%
	Not determined	12	39%
CD117	Pos	28	90%
	Focally pos	1	3%
	Neg	1	3%
	No data	1	3%
CD34	Pos	22	71%
	Focally pos	2	6%
	Neg	1	3%
	No data	6	19%
DOG1	Pos	15	48%
	Neg	6	19%
	Focally pos	4	13%
	No data	1	3%
	Not determined	5	16%
Necrosis	YES	9	29%
	NO	19	61%
	Not determined	3	10%
Initial stage	Locally advanced	4	13%
	Localized	19	61%
	Metastatic	8	26%
Radical surgery	YES	24	77%
	NO	7	23%
Vital status	ALIVE	24	77%
	DEAD	6	19%
	No data	1	3%
Site molecular analyses	Stomach	15	48%
	Ileum	6	13%
	Duodenum	1	3%
	Peritoneum	2	19%
	Liver	4	16%
	Jejunum	2	16%
	Small bowel	1	16%
Type of molecular analysis specimen	Surgery	22	48%
	Biopsy	2	13%
	Biopsy on relapse	4	3%
	FNAB	3	19%
Molecular status	KIT‐mutated	22	71%
	PDGFRA‐mutated	5	16%
	BRAF‐mutated	0	0
	SDH‐mutated	1	3%
	Quadruple WT	3	10%

In patients undergoing surgical resection, grading according to NIH [16] was performed. For each patient, six slides of 6 microns from the formalin fixed‐paraffin embedded (FFPE) block representative of the neoplasm were obtained. Neoplastic area was identified by a dedicated Pathologist (Paola Parente), and neoplastic cellularity in percentage was estimated on hematoxylin–eosin stained slides. All samples were handled in compliance with the Declaration of Helsinki (https://www.wma.net/what‐we‐do/medical‐ethics/declaration‐of‐helsinki/).

### 2.2 DNA Extraction and Targeted Sequencing

After manual microdissection of the neoplastic cells, DNA was extracted from FFPE tissues by using the QIAamp DNA Micro kit (Qiagen, Hilden, Germany), according to the manufacturer's instructions. DNA quantity and quality were assessed by the Qubit photometer (Thermo Fisher Scientific, Waltham, MA, USA). Two distinct panels were used for the analysis of tissue samples. The Oncomine DX Target Test (Thermo Fisher Scientific, Waltham, MA, USA), a commercially available CE‐IVD certified assay, was employed to analyze 35 genes, including *KIT*, *PDGFRA*, and *BRAF*. Additionally, a custom‐designed panel (IAD_202596_Spike‐In, Thermo Fisher Scientific) was used to assess the entire coding regions of *SDHA*, *SDHB*, *SDHC*, and *SDHD*. For the evaluation of germline mutations, we utilized a separate custom panel comprising 16 genes, provided by Agilent Technologies.

For each sample, 10 ng of dsDNA was used to prepare libraries with OncomineTM DX Target Test CE‐IVD KIT (Thermo Fisher Scientific). The Oncomine DX Target Test provides 269 amplicons covering the hotspot regions of 35 genes *(AKT1, ALK, AR, BRAF, CDK4, CTNNB1, DDR2, EGFR, ERBB2, ERBB3*, *ERBB4, ESR1, FGFR2, FGFR3, GNA11, GNAQ, HRAS, IDH1, IDH2, JAK1, JAK2, JAK3, KIT, KRAS, MAP2K1, MAP2K2, MET, MTOR, NRAS, PDGFRA, PIK3CA, RAF1, RET, ROS1, SMO*). The *SDHA*, *SDHB*, *SDHC*, and *SDHD* genes were analyzed by using an ION Ampliseq Custom panel (IAD_202596_Spike‐In) that provides 86 amplicons covering the whole sequence of the *SDH* genes and the *PDGFRA* gene.

Genetic variants were detected using the Ion Reporter Software v5.18.0 with high stringency settings, with a 5% allelic frequency cut‐off to exclude false‐positive results.

Germline analysis of the *SDHB* gene was performed on DNA obtained from whole blood extracted on the EZ1 Advanced Instrument. Genomic libraries were prepared on the Agilent Bravo, and enrichment was performed by the SureSelect system (Agilent). Libraries were sequenced on the MiSeq Desktop sequencer (Illumina) by using the MiSeq reagent Kit V3. The following genes were included in the panel: *APC, BMPR1A, CDH1, DICER, KIT, MLH1, MSH2, MSH6, MUTYH, NTHL1, PDGFRA, POLD1, POLE, PTEN, SDHB, STK11*. Genetic variants were detected by using the BWA version 0.7, GATK version 4, and ANNOVAR.

Mutations were verified in the Integrative Genomics Viewer (IGV) from the Broad Institute (http://www.broadinstitute.org/igv/), and their clinical relevance was evaluated by using ACMG/AMP standard guidelines for interpretation of genetic variants [14] and ASCO‐CAP guidelines for the interpretation and reporting of sequence variants in cancer [15].

### 2.3 In Silico Analyses

Pre‐computed pathogenicity predictions for the three unclassified mutations were retrieved from different indel‐centered in silico functional prediction software packages, including MutationTaster2, Cancer Genome Interpreter, CAPICE, FATHMM‐indel, MutPred‐Indel, and SIFT‐indel [17]. Moreover, the putative impact of candidate variants on *KIT* was evaluated by in silico structural biology techniques. First, the mutant proteins were modeled using ColabFold [18]. Then, the binding affinity difference (ΔΔGbinding = ΔGbinding_mut—ΔGbinding_wt) of each model to imatinib, compared to the wild‐type system, was evaluated by molecular docking with the GalaxyDockWEB tool [19], considering the ligand as flexible and the known binding pocket residues of the wild‐type protein as input to guide the docking.

## 3 Results

### 3.1 GISTs Molecular Characterization

Of the 31 patients, 27 (87%) showed at least one variant in genes included in the panels. As shown in Table 2, 22 patients showed at least one variant in the *KIT* gene (71%), five patients showed a mutation in *PDGFRA* (16%), and one patient showed a mutation in the *SDHB* gene (3%). Patient 22 showed a benign variant (p.Met541Leu) in *KIT* exon 10.

**TABLE 2:** Genetic variants detected in the patient cohort.

Patient	Mutated gene	Exon	Nucleotide variant	Protein variant	VAF	Type of tissue
1	*KIT*	11	c.1669 T>A	p.(Trp557Arg)	39%	Primary
2	*KIT*	11	c.1708_1728del	p.(Tyr570_Leu576del)	40%	Primary
3	*KIT*	11	c.1676 T>C	p.(Val559Ala)	45%	Primary
4	*KIT*	11	c.1697_1720del	p.(Asn566_Pro573del)	45%	Primary^a^
			c.1697_1720del	p.(Asn566_Pro573del)	40%	Relapse
5	*KIT*	13	c.1671_1676del	p.(Trp557_Val559delinsCys)	39%	Primary
6	*KIT*	13	c.1924A>G	p.(Lys642glu)	87%	Liver metastases
7	*KIT*	11	c.1669_1674del	p.(Trp557_Lys558del)	57%	Primary
8	*KIT*	11	c.1674_1724del	p. (Val559Gln575del)	11%–38%	Primary
		17	c.2448C>A	p.(Lys818Arg)	11%–38%	Metastases
9	*KIT*	11	c.1676 T>G	p.(Val559Gly)	45%	Primary
			c.1700A>G	p.(Asn567Ser)	45%	Primary
10	*KIT*	11	c.1271 T>C	p.(Leu576Pro)	67%	Relapse
11	*KIT*	11	c.1676 T>A	p.(Val559Asp)	25%	Primary
12	*KIT*	11	c.1676 T>G	p.(Val559Gly)	54%	Primary
13	*KIT*	11	c.1676 T>A	p.(Val559Asp)	48%	Primary
14	*KIT*	11	c.1670_1675delinsTCC	p.(Trp557_Val559delinsPhe)	50%	Primary
15	*KIT*	11	c.1665_1667delACA	p.Gln556del	41%	Primary
16	*KIT*	11	c.1676 T>A	p.(Val559Asp)	60%	Primary
17	*PDGFRA*	18	c.2525A>T	p.(Asp842Val)	89%	Primary
18	*PDGFRA*	14	c.1977C>A	p.(Asn659Lys)	42%	Primary
19	*PDGFRA*	14	c.1977C>A	p.(Asn659Lys)	36%	Primary
20	None					Liver metastases
21	*SDHB*	4	c.287‐1GC*	p.(?)	50%	Primary
22	*KIT*	10	c.1621A>C	p.(Met541Leu)	51%	Primary
	*NF1*	13	c.1466A>G*	p.(Tyr489Lys)		Primary
23	None					Primary
24	None					Primary
25	*PDGFRA*	18	c.2525A>T	p.(Asp842Val)	34%	Primary
26	*KIT*	11	c.(1727 T>C)	p.(Leu576Pro)	39%	Primary
27	*KIT*	11	c.1705_1728del	p.(Val569_Leu576del)	45%	Primary
28	*KIT*	11	c.1679 T>A	p.(Val560Asp)	37%	Primary
29	*KIT*	11	c.1679 T>A	p.(Val560Asp)	45	Primary
30	*PDGFRA*	18	c.2524G>T	p.(Asp842Tyr)	42%	Primary
31	*KIT*	11	c.1671_1676delGAAGGT	p.(Trp557_Val559delinsCys)	22%	Primary 2009^b^
		13	c.1961 T>C	p.(Val654Ala)	40%	Liver metastases 2024

^a^ An analysis carried out by another institution.

^b^ Retrospectively analyzed in 2024 to evaluate the presence of a secondary mutation.

As expected, *KIT*, *PDGFRA*, and *SDH* family mutations were mutually exclusive. Twenty‐one pathogenetic/likely pathogenetic variants were found in *KIT* exon 11 (70%), 2 in exon 13 (7%), and 1 in exon 17 (14%). Patient 9 showed double mutations in *KIT* exon 11 (p.Val559Gly and p.Asn567Ser). In the *PDGFRA* gene, three patients showed the p.Arg842Val (c.2525A>T, exon 18) (10%), and two patients showed mutations in exons 14 (7%).

Patient 8 was referred to our hospital at the time of disease progression. A *KIT* exon 11 deletion (c.1674_1724del; p.Val559_Gln575del), initially identified in the primary tumor at an external institution, was confirmed. In the recurrent tumor, we also detected an additional *KIT* exon 17 mutation (c.2448C>A; p.Lys818Arg). In Patient 31, a recurrent *KIT* exon 11 mutation (c.1671_1676del; p.Trp557_Val559delinsCys) was found in both the primary and recurrent tumors. Notably, an additional *KIT* exon 13 mutation (c.1961 T>C; p.Val654Ala) was identified exclusively in the recurrent sample.

Patient 21, affected by GIST, was initially referred for genetic counseling due to a family history suggestive of hereditary paraganglioma–pheochromocytoma syndrome (PGL/PCC). Genetic testing revealed a germline mutation in the *SDHB* gene, located at the intron–exon 4 boundary (c.287‐1G>C), which results in aberrant mRNA splicing. This variant was also present in the tissue sample and classified as pathogenic based on its location at a canonical splice site (±1–2), its absence from population allele frequency databases (dbSNP151, ExAC, gnomAD), and its annotation in ClinVar.

No pathogenic, likely pathogenic, or variants of uncertain significance (VUS) were identified in the *BRAF* gene in any of the analyzed cases. Overall, three patients did not harbor mutations in *KIT*, *PDGFRA*, *BRAF*, or *SDHA/B/C/D* and were therefore classified as quadruple‐negative (Table 1).

### 3.2 Classification of Rare *KIT* Exon 11 Mutations

Three rare variants were detected in *KIT* exon 11 c.1705_1728del (p.Val569_Leu576del), c.1665_1667delACA (p.Gln556del), and c.1697_1720del (p.Asn566_Pro573del). By interrogating the AACR‐Genie Consortium database (GENIE Cohort v13.0‐public) at https://genie.cbioportal.org/ on August 12, 2024, the p.Val569_Leu576_del was found in 5 GIST male individuals, with the deletion occurring in metastatic samples [20]. In contrast, no entries were found in the database for the p.Gln556del and p.Asn566_Pro573del variants. A review of the literature yielded a single report of p.Asn566_Pro573del, documented in the S1 of Palomba et al. [21]. None of the three variants was reported in allele frequency databases, and no evidence was available regarding their potential interaction with tyrosine kinase inhibitors (TKIs).

**FIGURE 1:**
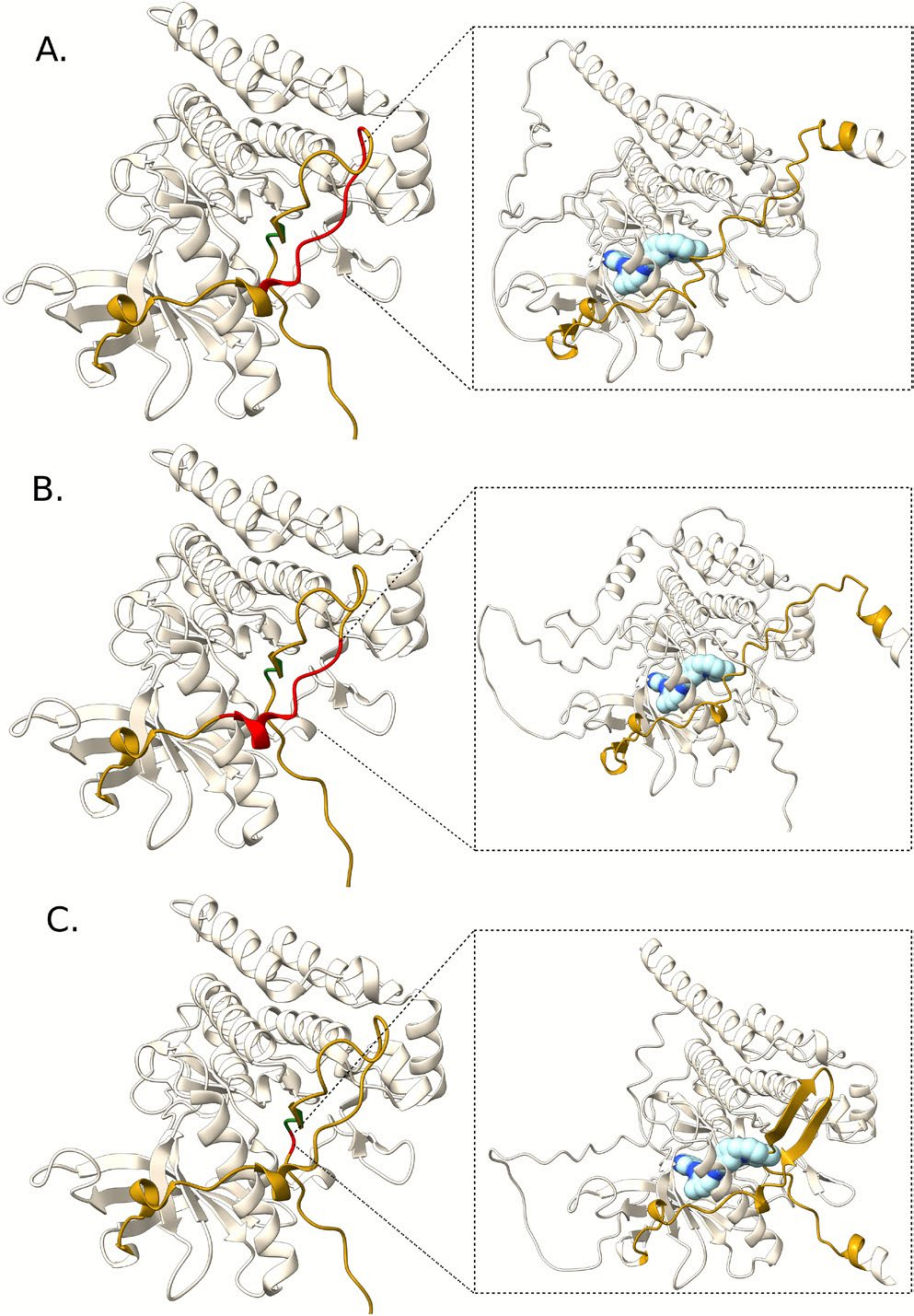
Structure of the KIT kinase domain (pdb_id: 1 t45). The juxtamembrane region (JM) is highlighted in gold, with residues 557–558 shown in green. Deleted regions—p.Asn566_Pro573del (A) p.Val569_Leu576del (B) and p.Gln556del (C)—are marked in red. On the right, 3D models of the KIT kinase domain are depicted with the respective deletions. Imatinib, shown in light blue, has been docked into its putative binding pocket by structural superimposition.

The first variant, identified in Patient 4, is an in‐frame deletion, c.1697_1720del, resulting in the eight‐residue deletion p.(Asn566_Pro573del) within the Juxta Membrane (JM) domain of the KIT protein. By modeling the mutant protein [14] (Figure 1A), we found that it deletes and disrupts a critical region responsible for the auto‐inhibitory function of the protein. This disruption results in the exposure of the catalytic binding pocket, which may enhance the binding of molecules (ΔΔG_binding_ = −13.579 Kcal/mol) such as imatinib to the ATP‐binding domain of KIT. This patient presented with an ileal primary tumor and histologically confirmed recurrence to lymph nodes, which is an uncommon site of metastasis in GISTs. The mutation was detected in both the primary tumor and the metastatic lesions, confirming it as a primary driver mutation. At the time of recurrence, the patient demonstrated preserved sensitivity to imatinib, achieving a progression‐free survival (PFS) of 10 months.

The second variant, c.1705_1728del, identified in Patient 27, consisted of the loss of eight residues (p.Val569_Leu576_del) within the JM of the KIT protein. This patient presented with a jejunum abdominal mass treated with surgery after neo‐adjuvant therapy with imatinib. In silico mutation prediction analysis indicates that the Val569_Leu576_del mutation determines a deletion in the critical region responsible for the autoinhibitory function of the KIT protein. This might lead to exposure of the catalytic binding pocket, facilitating interaction (ΔΔG_binding_ = −11.879 Kcal/mol) with molecules such as imatinib (Figure 1B).

The third variant, identified in Patient 15, is the c.1665_1667delACA p.(Gln556del). This patient was diagnosed with a large abdominal mass and peritoneal carcinomatosis and was sensitive to imatinib treatment even after dose reduction due to cutaneous toxicity. Nevertheless, for this variant, the deletion, highlighted in red in Figure 1C, seems not to impact the structural organization of the juxtamembrane region closed conformation, preserving its autoinhibitory role, with a binding affinity for imatinib comparable to that of the wild‐type protein (ΔΔG_binding_ = +3.925 Kcal/mol).

## 4 Discussion

GISTs represent a model for the paradigm shift towards targeted therapy of solid tumors. Indeed, the identification of activating mutations in *KIT* and *PDGFRA* receptors has driven the development of the imatinib TKI and the use of next‐generation TKIs, such as sunitinib, regorafenib, ripretinib, and avapritinib [7]. Molecular characterization of GISTs has recently acquired a crucial role in overall management, in both adjuvant and metastatic settings, predicting the sensitivity to targeted therapy, as well as prognosis.

The advent of Next Generation Sequencing (NGS) technologies has increased our ability to identify mutations in driver genes, broadening the spectrum of genetic alterations that may be detected. However, determining the clinical significance of genetic variants is a complex task that requires collecting and analyzing available evidence, followed by a formal classification based on that evidence [14, 15]. In this study, we report our real‐life experience in implementing NGS for the molecular characterization of GISTs.

We analyzed tissue samples from 31 GIST patients, profiling a total of 40 genes. Variants were detected in 27 patients (87%), each carrying at least one mutation in genes included in our panel. As expected, *KIT* showed the highest frequency of clinically relevant variants (71%), followed by *PDGFRA* (16%) and *SDHB* (3%). These findings are consistent with those reported by Fujii et al. [22], who performed a large‐scale genomic analysis of 144 GIST patients from the Japanese National Center for Cancer Genomics and Advanced Therapeutics (C‐CAT) database. Using the OncoGuide NCC Oncopanel System (114 genes) and FoundationOne CDx Cancer Genomic Profile (324 genes), they identified alterations in *KIT* (78%), *PDGFRA* (6%), and *SDHB* (6%). Additional mutations reported by Fujii et al. in genes not covered by our panel included *CDKN2A* (45%), *CDKN2B* (32%), *RB1* (11%), *STK11* (10%), *TP53* (9%), and *NF1* (4%). Notably, *CDKN2A/CDKN2B* mutations were associated with *KIT/PDGFRA*‐mutated GISTs. In line with our findings, no BRAF V600E mutations were observed in their cohort, and *SDHA/SDHB* mutations were detected exclusively in *KIT/PDGFRA* wild‐type tumors.

Approximately 40% of *KIT* and *PDGFRA* wild‐type GISTs display SDH deficiency. Among these, *SDHA* mutations are the most common and typically occur at the somatic level. Mutations in *SDHB*, *SDHC*, and *SDHD* are less frequent, and most are of germline origin. We identified a germline mutation in the *SDHB* gene (c.287‐1G>C), which results in aberrant mRNA splicing. This mutation was previously reported by Rinelli et al. [23] in a pediatric GIST case. Consistently, the patient in our study carrying this mutation was diagnosed at age 16, further supporting the hypothesis that germline *SDHB* mutations are associated with early‐onset GIST development.

Together with common primary and secondary mutations in *KIT* and *PDGFRA* genes, we detected three rare exon 11 mutations of unknown pathogenicity. All three mutations met the ACMG/AMP criteria for pathogenicity. Genetic variants located in exon 11 are generally associated with response to imatinib, with the majority of them involving codons 557/559 [24, 25, 26, 27]. From a structural point of view, despite an increased risk of relapse after surgery, deletions involving these codons are associated with an increased flexibility of the protein, promoting the open‐closed conformational transitions and, thus, allowing the interaction with ligands like imatinib. Recent studies have hypothesized that single mutations or deletions upstream or downstream of codons 557/559 might differently affect protein conformational transitions. Deletions downstream of codons 568–570 are infrequent, with less than three cases reported for each pathogenic variant [27]. Of the three rare genetic variants reported in this study, two are downstream of codons 557/559, whereas the third variant is located one codon upstream. Despite the different locations, all three mutations were associated with response to treatment with imatinib. Indeed, patient 4 showed preserved sensitivity to imatinib at the time of recurrence, patient 27 underwent surgery after neo‐adjuvant treatment with imatinib, and patient 15 showed a reduction of the abdominal mass even in the presence of a protein conformation that, according to in silico modeling, seems not to facilitate the interaction with small molecules like imatinib.

Recently, liquid biopsy has emerged as a promising, minimally invasive tool for detecting tumor‐specific mutations in GISTs via analysis of circulating tumor DNA (ctDNA) [10, 13]. This approach is particularly valuable when tissue is not accessible or when longitudinal monitoring is needed. However, its current clinical applicability remains limited. In a study by Mechahougui et al. [13]. ctDNA analysis showed good concordance with tissue profiling in patients with higher tumor burden, identifying *KIT* mutations in 89% and secondary resistance mutations in 55% of cases. Despite these encouraging findings, ctDNA detection remains challenging in patients with localized or low‐volume disease due to low ctDNA levels, and further limitations include inter‐platform variability and a lack of standardized protocols. Therefore, liquid biopsy should be considered a complementary tool rather than a replacement for tissue biopsy. Tissue‐based analysis remains the gold standard for molecular profiling of GISTs, offering high accuracy in detecting both primary and secondary mutations, along with essential histopathological context for guiding clinical decision‐making.

We are aware that our study has some limitations. The first is its retrospective design, which impairs the clinical data collected at baseline conditions. The second is the small number of cases, which makes it difficult to estimate how often rare variants occur and to understand how they relate to clinical and pathological features. Using public databases might help to increase the number of reported cases and variants, but these databases often do not include detailed clinical information or long‐term follow‐up data. This limits their usefulness for studying the long‐term effects of specific mutations. Since GIST is a rare disease, with an estimated incidence of about 0.0004% (4.2 cases per million people per year), as reported in a recent study [28], only large, prospective multicenter studies will be able to reliably assess the impact of specific *KIT* variants on long‐term outcomes. Finally, the short follow‐up duration in our study prevents us from fully understanding the prognostic and predictive value of the mutations we observed. For example, we cannot rule out that the preserved autoinhibitory function of the Gln556del variant might influence sensitivity to imatinib or other tyrosine kinase inhibitors over time.

Although the cost of next‐generation sequencing (NGS) has decreased in recent years, financial sustainability remains a major barrier to its widespread adoption, especially in underfunded settings. In Italy, molecular testing access is generally broader than in private insurance‐based systems, but regional disparities persist, particularly in under‐resourced areas. At our institution, NGS‐based molecular testing for GISTs is reimbursed by the Italian National Health System (SSN) when clinically indicated for diagnosis, prognosis, or treatment decisions in oncology. As IRCCS (Istituto di Ricovero e Cura a Carattere Scientifico), we benefit from national and regional funding supporting advanced molecular diagnostics. However, such support is often lacking in less specialized or underfunded centers. To ensure equitable access to precision oncology, reimbursement policies must be harmonized, cost‐effective testing promoted, and funding expanded in underserved regions.

In conclusion, the implementation of NGS testing has significantly expanded our understanding of cancer molecular pathogenesis, while simultaneously raising critical challenges regarding the interpretation of novel or rare variants in genes with potential clinical relevance. Since public databases predominantly rely on literature‐based data for genetic variant classification, it is essential for laboratories to share molecular findings alongside detailed clinical information. This collective knowledge can serve as a foundation for more precise and effective patient treatments, thereby advancing both research and clinical care.

## Author Contributions

**Maria Grazia Rodriquenz:** conceptualization, writing – original draft, writing – review and editing, data curation, methodology, supervision. **Barbara Pasculli:** conceptualization, investigation, writing – original draft, writing – review and editing, methodology, supervision. **Michelina Rendina:** conceptualization, investigation, writing – original draft, writing – review and editing, methodology. **Francesco Petrizzelli:** writing – review and editing, software, formal analysis. **Tommaso Mazza:** writing – review and editing, software, formal analysis. **Antonio Petracca:** writing – review and editing, formal analysis, data curation, validation. **Bartolomeo Augello:** investigation, writing – review and editing, formal analysis. **Michelina Coco:** investigation, writing – review and editing, methodology. **Teresa Balsamo:** investigation, methodology, writing – review and editing. **Anna Troiano:** investigation, methodology, writing – review and editing. **Magda Zanelli:** investigation, methodology, validation, writing – review and editing. **Evaristo Maiello:** validation, formal analysis, data curation, writing – review and editing, resources. **Paolo Graziano:** validation, formal analysis, data curation, resources. **Paola Parente:** investigation, writing – original draft, methodology, validation, formal analysis, data curation, supervision, resources, writing – review and editing. **Paola Parrella:** conceptualization, writing – original draft, validation, writing – review and editing, resources, supervision, data curation.

## Funding

This work was supported by the Italian Ministry of Health (MoH) “Ricerca Corrente 2025” and by Italian Ministry pf Health “5 × 1000 voluntary contributions”.

## Ethics Statement

This study was conducted according to the guidelines of the Declaration of Helsinki and approved by the Ethics Committee of Fondazione “Casa Sollievo della Sofferenza”, IRCCS 71013 San Giovanni Rotondo, Italy.

## Consent

Prior written informed consent was obtained from all patients.

## Conflicts of Interest

The authors declare no conflicts of interest.

## Supporting information




**Supplemental Table 1**: Clinicopathological Characteristics of the patient's cohort.

## Data Availability

The authors have nothing to report.
